# The effects of exogenous hormones on rooting process and the activities of key enzymes of *Malus hupehensis* stem cuttings

**DOI:** 10.1371/journal.pone.0172320

**Published:** 2017-02-23

**Authors:** Wangxiang Zhang, Junjun Fan, Qianqian Tan, Mingming Zhao, Ting Zhou, Fuliang Cao

**Affiliations:** 1 College of Forestry, Nanjing Forestry University, Nanjing, China; 2 Co-Innovation Center for Sustainable Forestry in Southern China, Nanjing Forestry University, Nanjing, China; 3 Yangzhou Crabapple Horticulture Company Limited, Yangzhou, China; 4 Suzhou Forestry Station, Suzhou, China; Institute of medical research and medicinal plant studies, CAMEROON

## Abstract

*Malus hupehensis* is an excellent *Malus* rootstock species, known for its strong adverse-resistance and apomixes. In the present study, stem cuttings of *M*. *hupehensis* were treated with three types of exogenous hormones, including indole acetic acid (IAA), naphthalene acetic acid (NAA), or green growth regulator (GGR). The effects and mechanisms of exogenous hormone treatment and antioxidant enzyme activity on adventitious root formation were investigated. The results showed that the apparent morphology of the adventitious root had four stages, including root pre-emergence stage (S_0_), early stage of root formation (S_1_), massive root formation stage (S_2_), and later stage of root formation (S_3_). The suitable concentrations of the three exogenous hormones, IAA, NAA and GGR, were 100 mg·L^-1^, 300 mg·L^-1^, and 300 mg·L^-1^, respectively. They shortened the rooting time by 25–47.4% and increased the rooting percentages of cuttings by 0.9–1.3 times, compared with that in the control. The dispersion in S_0_ stage was 3.6 times of that in the S_1_ stage after exogenous hormone application. The earlier the third critical point (P_3_) appeared, the shorter the rooting time and the greater the rooting percentage of the cuttings. During rhizogenesis, the activities of three antioxidant enzymes (POD, SOD, and PPO) showed an A-shaped trend. However, peak values of enzyme activity appeared at different points, which were 9 d before the P_3_, P_3_, and the fourth critical point (P_4_), respectively. Exogenous hormone treatment reduced the time to reach the peak value by 18 days, although the peak values of the enzymatic activities did not significantly changed. Our results suggested that exogenous hormone treatment mainly acted during the root pre-emergence stage, accelerated the synthesis of antioxidant enzymes, reduced the rooting time, and consequently promoted root formation. The three kinds of antioxidant enzymes acted on different stages of rooting.

## Introduction

*Malus hupehensis* (Pamp.) Rehd. is a major accession of *Malus* spp. and is widely distributed across China [1, 2]. *M*. *hupehensis* has been cultivated as an ornamental tree and utilized as medicine and food. Recently, it has also been considered as a prime rootstock species in the southern region of the China Yellow River Basin [3]. Although *M*. *hupehensis* is regarded as a triploid species with facultative apomixes [4, 5], it also presents a certain level of differentiation post-sowing. Therefore, it is critical that stem cutting propagation of *M*. *hupehensis* be developed and implemented.

Vegetative propagation using the stem cutting method can be considered as a post-operation root regeneration process. Once a stem is cut from its parent plant, its nutrient and water supply is lost. Under such conditions, the plant is exposed to oxidative stress, and its redox balance is destroyed [6]. How to increase cellular antioxidant resistance, restore redox balance, and promote adventitious root formation (ARF) are critical to stem survival [7, 8]. The ARF process generally consists of three stages: induction, initiation, and extension (including outgrowth within the stem and outgrowth from the stem) [9]. Exogenous hormone treatment is one of the major methods for improving ARF [10, 11]. The mechanism underlying the promotion of ARF by exogenous hormone treatment has been largely investigated. Several studies have shown that exogenous hormone treatment accelerates cell division, promotes synthesis of endogenous hormones (e.g., auxins, cytokines, and gibberellins) and salicylic acid, stimulates carbohydrate accumulation, and consequently induces ARF [8, 12–14]. Exogenous hormone treatment also increases peroxidase (POD) and polyphenol oxidase (PPO) activity, decreases the activity of IAAO and subsequently promotes root formation. The activity of POD and PPO continuously increases during the adventitious root induction and initiation phases, it gradually decreases during the extension phase, whereas the H_2_O_2_ profiles and IAAO activity are exactly the opposite of that of POD and PPO [15–17]. Therefore, changes in POD and PPO activity are often used as biochemical indicators for various stages of ARF [18–20]. However, most current studies have focused on microscopic cellular features prior to the formation of root primordia as well as other physiological and biochemical changes, whereas the importance of specific critical points such as adventitious root cortical breakthrough and massive root formation have not been established completely.

By using exogenous hormone treatment, this paper studied the dynamics of rooting of *M*. *hupehensis* cutting and the changes in the activity levels of antioxidant enzymes (POD, SOD, and PPO). The purposes were as follows: (1) To establish the critical points of apparent morphology in the rooting process; (2) To explore the effect of exogenous hormones on the rooting process; (3) To explore the rhythm correlation between rooting process and three antioxidant enzymes (POD, PPO, and SOD).

## Materials and methods

### Plant materials

Cultures of *M*. *hupehensis* stem cuttings were established under natural light conditions. The cutting matrix consisted of a perlite-vermiculite mix (2:1 vol). The cutting matrix depth was 15 cm. In February, stem cuttings were prepared from one-year-old parent plants of *M*. *hupehensis*. All the cuttings were 10–12 cm in length, 0.5–0.6 cm in diameter, and with 4–6 buds.

### Methods and measurements

#### Evaluation of the root regeneration process

A two-factor randomized experiment was performed. A total of 13 treatment groups using four different concentrations (100, 300, 500, or 700 mg·L^-1^) of IAA, NAA, or GGR (20% amino acids, 2% trace elements; Beijing Erbitux Biological Technology Co., Ltd., Peking, China.) and one water-treated control group were assessed in terms of morphological characteristics and rooting percentages. Each treatment consisted of 600 cuttings that were further divided into two groups, with each group comprising 300 cuttings.

Group 1 was used to observe the rooting process every 3 days after planting in which five cuttings were randomly selected for observation. The initial date of planting was designated as P_0_. The date of root emergence through the stem epidermis was identified as the root emergence day (P_3_). The date when the number of roots reached ≥ 3, root length measured ≥ 1 cm, and the lateral root started to appear was recorded as the day of massive root formation (P_4_). The date when the structured main lateral root system was formed and 80% of the root system changed to brownish red was denoted as the day of root system formation (P_5_). The root pre-emergence stage (S_1_) consisted the period from P_0_ to P_3_, the early stage of root formation (S_2_) included the period from P_3_ to P_4_, the root massive formation stage (S_3_) comprised the period from P_4_ to P_5_, and later stage of root formation contained the time after P_5_. Because no anatomical observations were made before the root per-emergence stage, for the consistency of the description of the rooting process, the day of root primordia initial cell formation in this stage was noted as P_1_’, and the time for formation of the root primordium was P_2_’ [9].

Group 2 was used for the analysis of the percentage of cuttings that underwent rooting. At 72 d post-planting, three subgroups were randomly chosen from the respective 13 treatment groups, and each subgroup consisted of 30 cuttings.

#### Measurement of antioxidant enzymes activity

Approximately 2,000 cuttings were subjected to treatment using 100 mg·L^-1^IAA or water to monitor the dynamic changes in the activities of POD, SOD and PPO during root regeneration. Cutting samples were collected at the day of planting, and at each 9-d interval. At each time point, three subgroups were randomly selected from each treatment group and each subgroup included 30 cuttings. The collected cuttings were rinsed with water, kept in an ice box. The phloem located 2 cm from the base of the cuttings were peeled, cut into pieces, and kept at −70°C until further testing.

To measure the activity of antioxidant enzymes, 0.2 g of each sample was weighed from the above mixture of tissues from each subgroup of every treatment with three replicates. The sample was ground in a mortar on ice with 6 mL of 0.05 mol·L^−1^ pre-cold phosphate buffer (pH 7.8) for enzyme extraction. The homogenate was centrifuged at 9,000 rpm for 20 min at 4°C, the pellet discarded, and the supernatant was used in the enzyme assay.

POD activity was determined according to the method of Tian et al. [21] based on the oxidation of guaiacol using H_2_O_2_. Briefly, 0.2 mL of the enzyme extract was added to a reaction solution containing 3.8 mL of 0.3% guaiacol potassium sulfonate and 0.1 mL of 0.3% H_2_O_2_. One unit of POD activity was defined as the increase in absorbance recorded at one OD value of A_470_ per min per gram fresh weight. Phosphate buffer was used as blank control.

SOD activity was determined according to the method of Giannopolitis and Ries [22], which measured its ability to inhibit the photochemical reduction of nitrobluetetrazoliumas. Briefly, 0.05 mL of the enzyme extract was added to a 2.95-mL reaction solution containing 0.3 mL of 20 μmol·L^−1^ riboflavin, 0.3 mL of 130 mmol·L^−1^ methionine, 0.3 mL of 750 μmol·L^−1^ NBT, 0.3 mL of 100 μmol·L^−1^ Na_2_EDTA, 1.5 mL of 0.05 mol·L^−1^ potassium phosphate buffer (pH 7.8) and 0.25 mL of distilled water. The reaction solution was placed under a 4,000 lx fluorescent lamp for 20 min, whereas the control sample was kept in dark. The solution was then placed in the dark for 5 min to stop the reaction. One unit of SOD activity was defined as the amount of enzyme that inhibited 50% of NBT and was expressed as unit per min per gram fresh weight. Phosphate buffer was used as blank control.

PPO activity was determined according to the method of Yan et al. [23]. Briefly, 0.5 mL of the enzyme extract was added to 4.5 mL of a reaction solution containing 3.5 mL phosphate buffer (pH 6.0), and 1 mL of 0.1 mol·L^−1^ catechol, mixed well, and incubated at 37°C for 10 min. Approximately 2 mL of 20% trichloroacetic acid was added to the mixture to stop the reaction, which was then followed by centrifugation at 6,000 rpm for 10 min. One unit of PPO activity was defined as the increase in absorbance recorded at one OD value of A420 per min per gram fresh weight. An inactivated enzyme solution was used as blank control.

### Data analysis

Statistical analysis was performed using SAS 8.1 software (SAS Institute). ANOVA was performed for each variable and the means were compared by using Duncan’s multiple range test at a significance level of 0.05 or 0.01. A graph was prepared by using Origin Pro 8 software.

The effect of exogenous hormones on the rooting stage was analyzed, and expressed as the dispersion of the rooting stage length between the treatment and the control. The following formula was used:

$$\sigma^{\prime }=\sqrt{\frac{\sum_{i=1}^{12}{\left(\chi_{i}-\chi_{0}\right)}^{2}}{12}}$$

where *x*_i_ represents the time to root in each treatment group, *x*_0_ represents the time to root in the control group.

## Results

### Observation of the morphological characteristics of *Malus hupehensis* during rhizogenesis of cuttings

**Fig 1:**
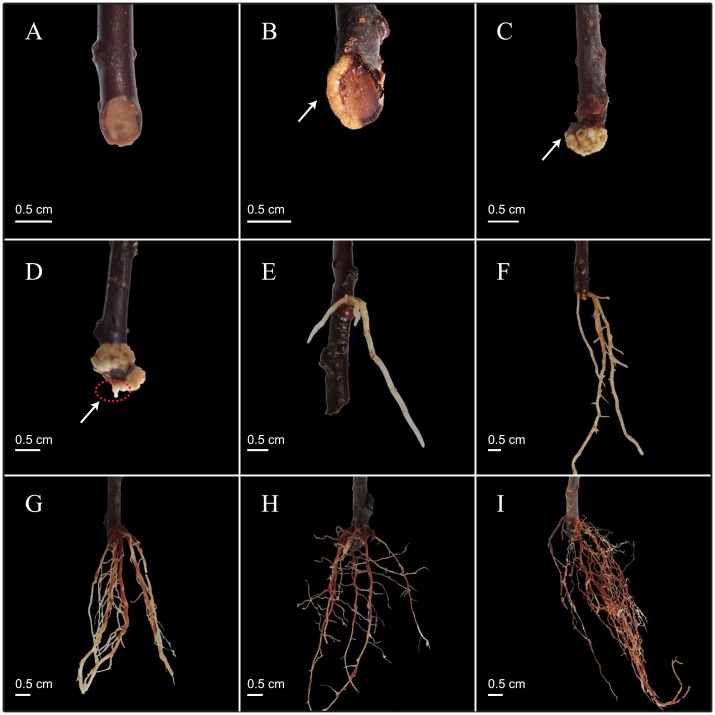
Dynamic changes in apparent morphology during rhizogenesis of *Malus hupehensis* cuttings (with IAA 100 mg•L^−1^ treatment). A–I were after IAA hormone treatment of cuttings lasting 0 d, 9 d, 18 d, 27 d, 36 d, 45 d, 54 d, 63 d, and 72 d, respectively.

The dynamic observation graph and explanation of rooting of cutting of *Malus hupehensis* treated with IAA 100 mg·L^−1^ are shown in Fig 1 and Table 1. Results showed that the apparent morphology of the adventitious root had four stages (pre-emergence, early stage of root formation, massive root formation, and later stage of root formation), and each stage had distinct morphological characteristics. The characteristic of the root pre-emergence stage was the formation and enlargement of the callus (Fig 1A,1B and 1C). The characteristic of the early stage of root formation was the emergence of young adventitious roots, which were located at the bark of the cutting base (about 0–2 cm away from the incision) rather than callus (Fig 1D and 1E). This showed that the rooting type of cutting of *M*. *hupehensis* was the phloem-rooting type. The most important characteristic of the massive root formation stage was the increase in the number of adventitious roots and the emergence of lateral roots (Fig 1F and 1G). The most important characteristic of later stage of root formation was the formation of the structured main lateral root system (Fig 1H and 1I).

**Table 1:** Morphological characteristics of *Malus hupehensis* during rhizogenesis of cuttings.

Rooting stage	Days after planting (d)	Description of appearance.	Function and significance	Critical Points
**Root pre-emergence stage (S**_**0**_**)**	0	Incision smooth, fresh.	Physiological induction and formation of root primordia.	P_1_’, P_2_’
	9	In the 1/4 to 1/2 of the edge of the incision, milk yellow callus formed with circular distribution.		
	18	Milk yellow callus expanded to beyond 2/3 of the edge of the incision.		
**Early stage of root formation (S**_**1**_**)**	27	Milky white adventitious roots emerged from the epidermis.	The formation and recovery of root absorption function is an important indicator of the survival rate of cuttings.	P_3_
	36	More milky white adventitious roots emerged from the epidermis and the length of the root increased.		
**Massive root formation (S**_**2**_**)**	45	The differentiation of the main roots and the lateral root began to form; the number of main roots with length ≥ 1 cm was more than 3; the base of the root gradually took on a pale reddish brown color; level one lateral roots began to appear.	The root absorption function was significantly enhanced, and the ability of root regeneration was strong at transplanting stage, with certain stress resistance and adaptability.	P_4_
	54	The number of adventitious roots increased further; about 50% of the roots turned into pale brown red.		
**Root post-emergence stage (S**_**3**_**)**	63	The structured dominant lateral root system has been formed, and about 80% of the roots changed to brownish red.	Stress resistance and adaptability were strong, but the ability of root regeneration decreased when transplanted decreased.	
	72	A well-developed root system has developed with a dark brownish red color.		

P_1_’ represents the date of root primordia initial cell formation; P_2_’ represents the date for formation of the root primordium; P_3_ represents the date of adventitious root emergence through the stem epidermis, P_4_ represents the date of massive root formation.

### The effects of exogenous hormone treatment during rhizogenesis of cuttings

**Fig 2:**
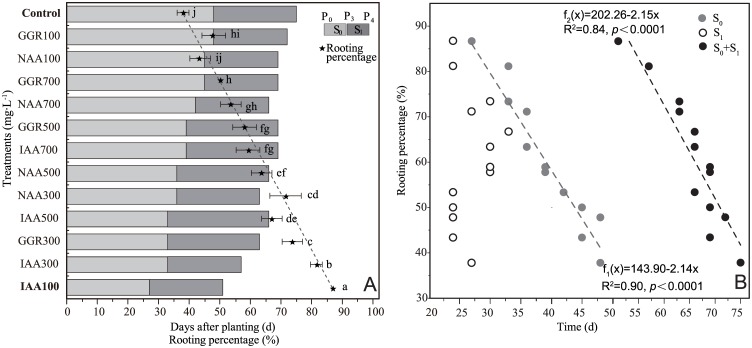
Relationship between rooting stage of *Malus hupehensis* cuttings and rooting percentage. A: Effects of different exogenous hormones on rooting percentage and rooting stage of *Malus hupehensis* cuttings, B: Correlation between different rooting stages and rooting percentage. P_0_ represents the date when cuttings were planted. S_1_ is the root pre-emergence stage, which represents the time from P_0_ to P_3_; and S_2_ is the early stage of root formation, which represents the time from P_3_ to P_4_. Rooting percentage data was transformed using arc sine after square-root transformation for ANOVA. Different lower case letters behind each rooting data indicate significant differences at p ≤ 0.05 by using Duncan’s test.

As shown in Fig 2A, the type and concentration of hormone had a significant effect on rooting percentage of cutting and rooting time of cutting (*P* < 0.0001). The suitable concentrations of three exogenous hormones (IAA, NAA, and GGR) for rooting were 100 mg·L^-1^, 300 mg·L^-1^, and 300 mg·L^-1^, respectively, the rooting percentages were 2.29, 1.88, and 1.94 times the control values, respectively, and the durations of the rooting formation (S_1_) were shorted by 47.4%, 25.0%, and 31.3%, respectively(Fig 2A). There was a significant negative correlation between rooting time of cutting and cutting rooting percentage (Fig 2B). During the S_0_ and S_0_+S_1_ stages, the relationship function between rooting time of cutting and cutting rooting percentage were f_1_(x) = 143.90 − 2.14x (R^2^ = 0.90, *P* < 0.0001) and f_2_(x) = 202.26 − 2.15x (R^2^ = 0.84, *P* < 0.0001), respectively, indicating that the shorter the rooting time, the greater the rooting percentage. However, results showed that the slopes of the two linear functions f_1_(x) and f_2_(x) were almost equal. In fact, no significant correlation was observed between the S_1_ stage and the cutting rooting percentage (Fig 2B). Based on the rooting data presented in Fig 2A, the dispersion of S_1_ (σ’ = 11.6 days) was determined to be obviously longer (3.6 times) relative to that of S_2_ (σ’ = 3.2 days).

### Changes in enzyme activities during rhizogenesis

**Fig 3:**
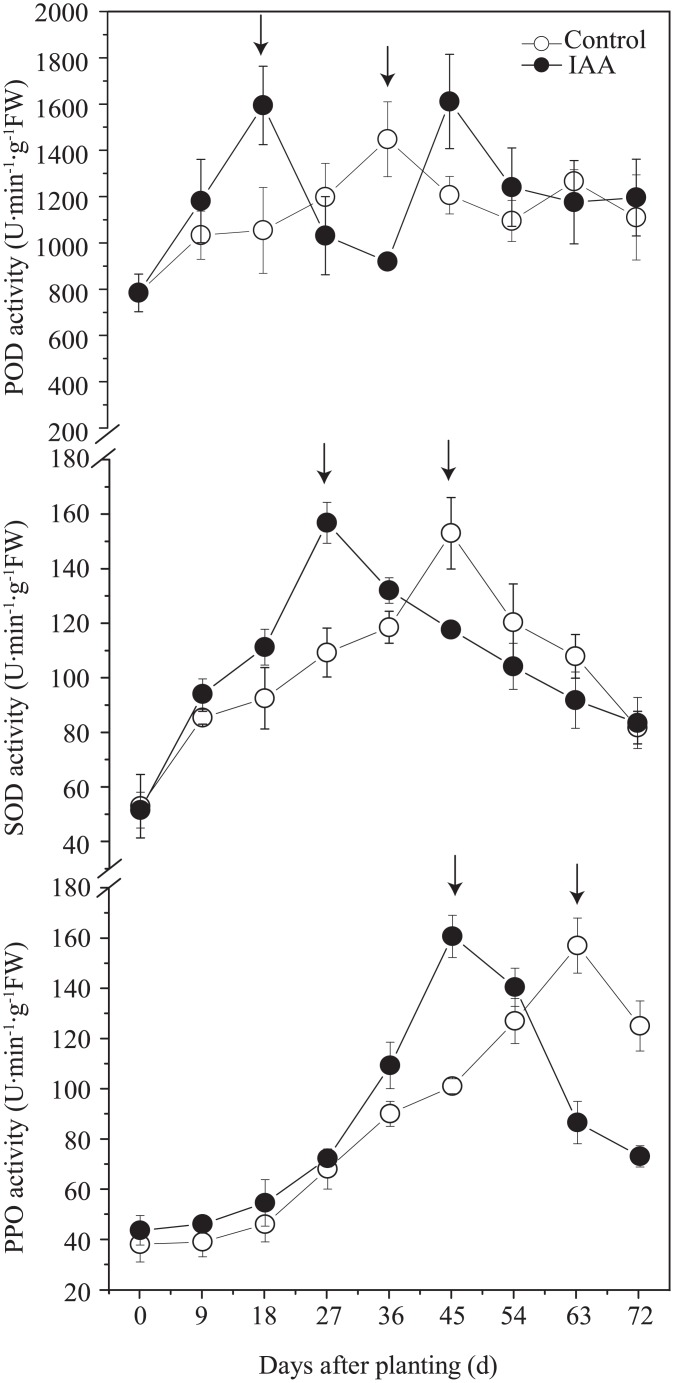
Changes in POD, SOD, and PPO enzyme activities during rhizogenesis in cuttings of *M*. *hupehensis*.

During the rooting process, the enzymatic activities of POD, SOD, and PPO in the phloem tissue of the cuttings followed an A-shaped trend, wherein it initially increased, reached a peak value, and then decreased (Fig 3). However, the time to reach its peak value varied among the three enzymes (as indicated by the arrow in Fig 3). The POD activity peaked at 9 days before the emergence of the adventitious roots, the time to reach the highest SOD activity synchronized well with the emergence time of the adventitious roots, whereas PPO activity peaked at 18 days after the development of adventitious roots (which synchronized well with root massive emergence).

The characteristic values (peak values and simultaneous values) of POD, SOD, and PPO were extracted from Fig 3 and presented in Table 2. Table 2 shows that exogenous hormone treatment significantly increased the synthesis rate of POD (*P* = 0.02), SOD (*P* = 0.0007), and PPO (*P* = 0.0002), and the time to reach its peak value was reduced to 18 days as compared to that observed in the control group. In addition, the peak values of POD, SOD, and PPO were 51.3%, 51.3%, and 75.1%, respectively, higher than the simultaneous value of the control group. However, the peak values of POD, SOD, and PPO in the exogenous hormone groups and the control group were not significantly different (*P* > 0.05).

**Table 2:** Characteristic values (peak and simultaneous values) of POD, SOD, and PPO in the phloem tissue of exogenous hormone-treated cuttings during rhizogenesis.

Treatment	POD activity/ (U·min^−1^·g^−1^FW)		SOD activity / (U·min^−1^·g^−1^FW)		PPO activity(U·min^−1^·g^−1^FW)	
	Peak value	Simultaneous Value	Peak Value	Simultaneous Value	Peak Value	Simultaneous Value
**Control**	1,447.50 ± 161.60	1,053.75 ± 185.65	153.01 ± 13.12	109.00 ± 9.00	156.5 ± 10.93	101.25±3.44
**IAA**	1,593.75 ± 169.34	1,593.75 ± 169.34	165.27 ± 4.766	165.27 ± 4.766	177.25 ± 9.56	177.25±9.56
***Y* (%)**	10.10^ns^	51.25*	8.01^ns^	51.28**	13.26^ns^	75.06**

Data are expressed as the mean ± SD (N = 90). Simultaneous value, representing the values measured in the control group at the same time when the peak values were reached in the IAA-treated group; Y = (IAA − Control)/Control × 100%.

ns: nonsignificant,

* *P* < 0.05,

**P < 0.01.

### Synergistic relationship between morphogenesis and endogenous substances during rhizogenesis

**Fig 4:**
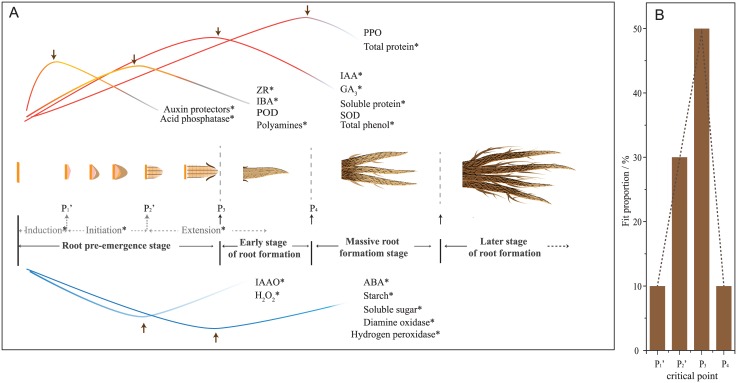
Fitting relationship between the dynamic change in endogenous substances and the apparent critical points during rhizogenesis. A: Schematic change in the levels of endogenous substances of cuttings during the formation of adventitious roots. B: The fit proportion between endogenous substances and the apparent critical points. P_1_’ represents the establishment of primordial cells in the adjacent cambium; P_2_’ represents adventitious root primordia and meristem initiation; P_3_ represents adventitious roots emergence through the epidermis of the stem; P_4_ represents the number of root ≥ 3, average root length ≥ 1 cm and the lateral root started to appear. *Adapted from references [15, 24–27]. Abbreviation: GA_3_, gibberellic acid; IBA, indole butyric acid; ZR, zeatin riboside.

In order to analyze the synergistic relationship between morphogenesis and the endogenous substances in the rooting process of cuttings, four critical morphological points closely related to rooting were assessed: initial formation of root primordial cells (first critical point), root primordium formation (second critical point), adventitious root breakthrough epidermis (third critical point), and adventitious root massive occurrence (fourth critical point). According to the statistical analysis of the three key enzymes in the study and the data in the literature [15, 24–27], results showed there were obvious differences between the fit proportion of the four key critical points and the anabolic rhythm of endogenous substances (peak/valley) during rhizogenesis (Fig 4A). The endogenous hormones, enzymes, and carbohydrates associated with rooting were mainly fit at the second and third critical points (fit proportion of 30% and 50%, respectively) (Fig 4B). Before the third critical point, the fit proportion continued to rise but declined sharply once reaching the fourth critical point (Fig 4B).

## Discussion

### Establishment of key critical points of apparent morphology in the rooting process of cuttings

Adventitious root formation (ARF) is a complicated process involving morphological, physiological, and biological changes [19, 28]. Based on observed anatomical changes [9], the ARF process was divided into three stages: induction (primordial initial cell establishment and pith development with cell de-differentiation followed by cell division), initiation (primordia and meristems initiate), and extension (including outgrowth in stems and outgrowth from stems) (Fig 4A). In the present study, based on the dynamic observation of the process of root formation, ARF was divided into root pre-emergence stage (S_0_), early stage of root formation (S_1_), massive root formation stage (S_2_), and later stage of root formation (S_4_) (Fig 4A). The major differences between these two staging system are that the S_0_ stage included the stage of induction, initiation, and outgrowth in stem of the former staging system, whereas the outgrowth from stem stage of the former staging system was further divided into S_2_, S_3_ and S_4_ in our new staging system. To elucidate the mechanisms and process of ARF, the former staging system is more focused on the microscopic observation at the cellular and tissue level using initial formation of root primordial cells and the formation of root primordia as the first and second critical points, respectively. On the other hand, our proposed staging system mainly concentrates on morphological observations of plant organs levels using adventitious root cortical breakthrough and massive root formation as the third and fourth critical points, respectively (Fig 4A). Most current studies pay more attention to the first and second critical points in the early stage of ARF while neglecting the third and fourth critical points in the late stage of ARF. However, the third and fourth critical points are highly significant to the survival rate of cuttings and transplants. The third critical point (adventitious root cortical breakthrough) indicates the initiation of root absorption function recovery, which is an important parameter for the evaluation of the survival rate of cutting, whereas the fourth critical point (massive root formation) represents the recovery of root absorption function and an increase in adaptiveness, which are important parameters affecting transplant survival rate.

In this study, at suitable concentrations, all the three exogenous hormones significantly promoted rooting of the cuttings (the rooting percentage was 1.88–2.29 times higher than that of the control, and the rooting time of S_0_ was shortened by 25.0–47.4%) (Fig 2A). Due to the discovery of the significant negative correlation between the timing of the third critical point (P_3_) appearance and cutting rooting percentage, the earlier the P_3_ appeared, the greater the rooting percentage. Of course, there was a different correlation between different rooting stages and cutting rooting percentage. There was a significant correlation between S_0_ stage and cutting rooting percentage (R^2^ = 0.90, *P* < 0.0001), but there was no correlation in the S_1_ stage (Fig 2A and 2B). In addition, we found that the dispersion of S_0_ stage was significantly longer than that of S_1_ stage, indicating the rooting effects of exogenous hormone treatment during S_0_ stage (the period before P_3_) was greater than during S_1_ stage (the period from P_3_ to P_4_). This might be related to the higher (50%) fit proportion at P_3_ (five times to that at the P_4_) (Fig 4B). In this way, the third critical point (adventitious root penetrating epidermis, P_3_) was crucial for rooting of cutting.

Many researchers believe that the root primordium induction stage is the key stage of cutting rooting [9, 29]. However, this assumption lacks statistical supporting data. Although this study did not include anatomical observation, the statistical results supported this view because the fit proportion of the second critical point (root primordia formation, P_2_) was as high as 30% (Fig 4B).

### The effects of three types of antioxidant enzymes (POD, PPO, and SOD) on different stage during root formation in *M*. *hupehensis* cuttings

Once the stems are cut from the parent plant, its nutrient and water supplies are cut off. The stems are thus under stress prior to the formation of adventitious roots. How to improve its resistance to stress and to reduce the time needed for root formation are critical for the survival of the cuttings. Antioxidant enzymes such as POD, PPO and SOD not only play important roles in a plant’s antioxidant defense [30, 31], but also affect the formation and development of adventitious roots [19, 29, 32]. In the present study, we found that the synthesis of these three enzymes varied among stages of the ARF process. The peak activity of POD was observed at 9 days before the third critical point, and the peak activity of SOD synchronized with the third critical point, whereas the peak activity of PPO synchronized with the fourth critical point (Figs 3 and 4). The observed differences might be explained by the fact that these three types of antioxidant enzymes promote ARF using distinct mechanisms.

The cell wall is an important defense barrier against pathogen invasion and expansion [33]. Increased lignin synthesis and extensin accumulation facilitate cell wall formation and improve its strength [34]. Increased POD activity promotes the biosynthesis of lignin and phellem layer; it also promotes the production of iso-2-tyrosin in hydroxyproline-rich glycoproteins (HRGP; extensin) [15, 33, 35]. Therefore, a moderate reduction in POD-type enzymes activity could decrease cell wall strength and consequently promote cell division, expansion, and plant growth, whereas an increase in POD-type enzyme activity increases the resistance of cells to stress [17, 34, 36]. The present study determined that the POD activity steadily and gradually increased during the early stages of root formation in cuttings and peaked at 9 days before the third critical point. The increase in POD activity at the early stage facilitates in scavenging for H_2_O_2_ molecules, increases cell wall strength, and subsequently increases resistance to stress. At a later stage, POD activity decreases, which in turn facilitates cell expansion and growth.

PPO promotes cell division, differentiation, as well as root primordia formation and development [37]. PPO also accelerates the formation of IAA-phenolic compounds and consequently promotes ARF [15, 17, 38]. The present study showed that PPO activity continuously increases before the emergence of the fourth critical point and peaks synchronously with the fourth critical point. This result indicates that continuous increases in PPO activity promote ARF throughout the entire process of induction to development.

As an important antioxidant defense line, SOD catalyzes the dismutation of excess O_2_^·−^ into O_2_ and H_2_O_2_, which can be further catalyzed by POD and other enzymes to form H_2_O and O_2_, and therefore prevent the cells from compositional, structural, as well as functional damages caused by free oxygen radicals [39, 40]. Increased SOD activity can improve the defense of cuttings against stress by scavenging reactive oxygen [41]; however, the effects of SOD on root formation of cuttings have not been reported. The present study showed that SOD activity continuously increases before the third critical point and peaks synchronously with the third critical point. This finding suggests that a continuous increase in SOD activity mainly occurs prior to the formation of adventitious roots. Before root formation, cuttings are exposed to stress, and SOD activity continuously increases, which in turn enhances the resistance of cuttings against stress. After adventitious root emergence, the absorption function of the roots is recovered, thereby relieving the plant from stress, whereas SOD activity starts to decrease.

Exogenous hormone treatment significantly accelerated the synthesis of antioxidant enzymes (e.g. POD, PPO, and SOD), with the time to peak reduced to 18 days (Fig 3), thereby decreasing the time to root formation. However, exogenous hormone treatment did not change the peak activity of the enzymes (Table 2), thereby suggesting that ARF requires a certain amount of endogenous enzyme activity. However, this hypothesis requires further investigation.

## Conclusion

(1) In the rooting process of *Malus hupehensis* cutting, the apparent morphogenesis included four stages: root pre-emergence (S_0_), early stage of root formation (S_1_), massive root formation (S_2_), and later stage of root formation (S_3_). (2) At suitable concentrations, all the three exogenous hormones promoted rooting of the cuttings (the rooting percentage was 1.88–2.29 times higher than that of the control, and the rooting time of S_0_ was shortened by 25.0–47.4%). (3)Exogenous hormones mainly acted in S_0_ stage (the period before adventitious root penetrating epidermis, P_3_). The earlier P_3_ appeared, the shorter the rooting stage and the higher the percentage of the cuttings that undergo rooting. Third critical point was critical in rooting of cutting. (4) Exogenous hormone treatment significantly accelerated the synthesis of antioxidant enzymes (POD, SOD, and PPO). However, the three types of antioxidant enzymes acted on different rooting stages. The peak activity of POD was observed before the third critical point, and the peak activity of SOD synchronized with the third critical point, whereas the peak activity of PPO synchronized with the fourth critical point.

## Supporting information

S1 Table Effects of different exogenous hormones on rooting time and rooting percentage of *Malus hupehensis* cuttings. P_0_, the starting date; P_3_, the root emergence date; P_4_, the massive root formation date; S_0_, the root pre-emergence stage (P_0_-P_3_); S_1_, early stage of root formation (P_3_-P_4_).
(DOCX)

S2 Table Changes in POD, SOD, and PPO enzyme activities during rhizogenesis of *Malus hupehensis* cuttings. (DOCX)
