# Supplementary material for: The effects of exogenous hormones on rooting process and the activities of key enzymes of Malus hupehensis stem cuttings
Source: PLoS One. 2017 Feb 23;12(2):e0172320. doi: 10.1371/journal.pone.0172320 (PMC5322878; doi:10.1371/journal.pone.0172320)
Supplement: S1 Table — P0, the starting date; P3, the root emergence date; P4, the massive root formation date; S0, the root pre-emergence stage (P0-P3); S1, early stage of root formation (P3-P4). (DOCX) [file pone.0172320.s001.docx]

**The effects of exogenous hormones on rooting process and the activities of key enzymes of *Malus hupehensis* stem cuttings**

Wangxiang Zhang, Junjun Fan, Qianqian Tan, Mingming Zhao, Ting Zhou, Fuliang Cao

**S1 Table. Effects of different exogenous hormones on rooting time and rooting percentage of *Malus hupehensis* cuttings.** P_0_, the starting date; P_3_, the root emergence date; P_4_, the massive root formation date; S_0_, the root pre-emergence stage (P_0_-P_3_); S_1_, early stage of root formation (P_3_-P_4_).

| Treatments | | P_0_ | P_3_ | P_4_ | S_0_ | S_1_ | Rooting percentage (%) |
| --- | --- | --- | --- | --- | --- | --- | --- |
| Control (water) (X_0_) | | 0 | 48th | 75th | 48 | 27 | 37.8 ± 1.9 |
| IAA concentration (mg·L^-1^) | 100 (X_1_) | 0 | 27th | 51st | 27 | 24 | 86.7 ± 0.0 |
|  | 300 (X_2_) | 0 | 33rd | 57th | 33 | 24 | 81.1 ± 1.9 |
|  | 500 (X_3_) | 0 | 33rd | 66th | 33 | 33 | 66.7 ± 3.3 |
|  | 700 (X_4_) | 0 | 39th | 69th | 39 | 30 | 58.9 ± 3.8 |
| NAA concentration (mg·L^-1^) | 100 (X_5_) | 0 | 45th | 69th | 45 | 24 | 43.3 ± 3.3 |
|  | 300 (X_6_) | 0 | 36th | 63rd | 36 | 27 | 71.1 ± 5.1 |
|  | 500 (X_7_) | 0 | 36th | 66th | 36 | 30 | 63.3 ± 3.3 |
|  | 700 (X_8_) | 0 | 42nd | 66th | 42 | 24 | 53.3 ± 3.3 |
| GGR concentration (mg·L^-1^) | 100 (X_9_) | 0 | 48th | 72nd | 48 | 24 | 47.8 ± 3.8 |
|  | 300 (X_10_) | 0 | 33rd | 63rd | 33 | 30 | 73.3 ± 3.3 |
|  | 500 (X_11_) | 0 | 39th | 69th | 39 | 30 | 57.8 ± 3.8 |
|  | 700 (X_12_) | 0 | 45th | 69th | 45 | 24 | 50.0 ± 0.0 |
| Dispersion $\sigma'$=$\sqrt{\frac{\sum_{i=1}^{12} \text{（}\chi_{\text{i}}-\chi_{0}\text{）}^{2}}{12}}$ | |  |  |  | 11.6 | 3.2 |  |
