# Supplementary material for: The effects of exogenous hormones on rooting process and the activities of key enzymes of Malus hupehensis stem cuttings
Source: PLoS One. 2017 Feb 23;12(2):e0172320. doi: 10.1371/journal.pone.0172320 (PMC5322878; doi:10.1371/journal.pone.0172320)
Supplement: S2 Table — (DOCX) [file pone.0172320.s002.docx]

**The effects of exogenous hormones on rooting process and the activities of key enzymes of *Malus hupehensis* stem cuttings**

Wangxiang Zhang, Junjun Fan, Qianqian Tan, Mingming Zhao, Ting Zhou, Fuliang Cao

**S2 Table. Changes in POD, SOD, and PPO** **enzyme activities during rhizogenesis of *Malus hupehensis* cuttings.**

| Enzymes | Days after planting (d) | Enzyme activities (U·min^-1^·g^-1^FW) | | | | | |
| --- | --- | --- | --- | --- | --- | --- | --- |
|  |  | CK | | | IAA-treatment | | |
|  |  | set1 | set2 | set3 | set1 | set2 | set3 |
| SOD | 0 | 39.68 | 61.61 | 57.42 | 39.68 | 61.613 | 57.42 |
|  | 9 | 83.55 | 84.52 | 88.07 | 104.19 | 102.29 | 88.39 |
|  | 18 | 96.77 | 79.68 | 100.97 | 111.61 | 115.81 | 122.58 |
|  | 27 | 119.36 | 106.13 | 102.26 | 161.61 | 170.65 | 163.55 |
|  | 36 | 120.32 | 111.94 | 123.23 | 137.10 | 138.71 | 140.65 |
|  | 45 | 151.61 | 166.77 | 140.65 | 118.71 | 115.16 | 136.45 |
|  | 54 | 132.90 | 105.16 | 122.90 | 115.81 | 102.90 | 108.71 |
|  | 63 | 103.55 | 102.90 | 117.10 | 106.77 | 99.36 | 81.613 |
|  | 72 | 87.742 | 75.81 | 81.61 | 86.45 | 97.10 | 77.419 |
| POD | 0 | 877.50 | 731.25 | 742.50 | 885.00 | 735.00 | 750.00 |
|  | 9 | 967.50 | 1153.13 | 978.75 | 1395.00 | 1080.00 | 1065.00 |
|  | 18 | 1260.00 | 900.00 | 1001.25 | 1785.00 | 1455.00 | 1545.00 |
|  | 27 | 1366.88 | 1108.13 | 1119.38 | 1230.00 | 945.00 | 930.00 |
|  | 36 | 1631.25 | 1383.75 | 1327.50 | 900.00 | 945.00 | 915.00 |
|  | 45 | 1113.75 | 1265.63 | 1237.50 | 1800.00 | 1635.00 | 1395.00 |
|  | 54 | 1046.25 | 1198.13 | 1040.63 | 1065.00 | 1410.00 | 1245.00 |
|  | 63 | 1305.00 | 1209.38 | 1282.50 | 1380.00 | 1065.00 | 1080.00 |
|  | 72 | 995.63 | 1321.88 | 1012.50 | 1365.00 | 1035.00 | 1185.00 |
| PPO | 0 | 30.00 | 42.00 | 41.25 | 30.00 | 42.00 | 41.00 |
|  | 9 | 40.50 | 43.50 | 31.50 | 39.00 | 43.00 | 41.00 |
|  | 18 | 39.75 | 45.00 | 53.25 | 41.50 | 47.00 | 63.00 |
|  | 27 | 72.00 | 73.50 | 58.50 | 72.00 | 68.50 | 76.50 |
|  | 36 | 86.25 | 96.00 | 87.75 | 111.00 | 128.50 | 108.00 |
|  | 45 | 105.00 | 100.50 | 98.25 | 188.50 | 171.00 | 172.50 |
|  | 54 | 136.50 | 125.25 | 119.25 | 143.50 | 159.00 | 157.50 |
|  | 63 | 155.25 | 146.25 | 168.00 | 97.500 | 91.50 | 78.00 |
|  | 72 | 132.00 | 129.00 | 114.00 | 76.00 | 76.50 | 67.00 |
